# Monthly Summary

**Published:** 1875-02

**Authors:** 


### MONTHLY SUMMARY.

---

*On the Treatment of Wounds.*—Dr. G. M. Humphrey, at the recent meeting of the British Medical Association, presented [*British Medical Journal,* Sept. 19, 1874] a paper upon this subject. Cut surfaces, he said, seldom fail to adhere together and grow together—that is, to unite by first intention—if they be kept in apposition, however much they may have been exposed to air, as in wounds of the face, scalp, and fingers, where this may commonly be done. This, therefore, is the great desideratum. The obstacle to it is the accumulation of bloody-fluid in the wound keeping the surfaces apart, decomposing, and causing suppuration. No precautions hitherto have assured against this. Anything giving that assurance would be a most valuable discovery. The decomposition may be lessened sometimes, though not commonly prevented, by antiseptics, but fail to provide for escape by drainage. Professor Humphry had long adopted the plan, which he had brought before the Association at the Cambridge meeting in 1864, of approximating the edges by sutures, and leaving the wound quite uncovered and dry. No dressing being used, the pain of removing and re-applying plasters, etc., is avoided. This, combined with some antiseptic or blood-coagulator to the surface of the wound, and a space left for escape of bloody-fluid, was, he believed, the mode most likely, on the whole, to promote quick union of large deep wounds. He did not think that torsion of vessels or carbolized ligatures presented much advantage over the hemp ligature. A splint was an adjunct that might well be oftener used. He had tried the hydrate of chloral [five to eight grains to the ounce of water,] and found it a valuable disinfectant, and recommended others to try it. In the case of wounds for the removal of cancer, he had for many years encouraged suppuration, and tried to retard healing, believing that this tended to delay the return of the disease. In some cases, he had inserted issues at the time of the operation or subsequently, and he thought with benefit. He believed the administration of opiates to be generally prejudicial in treatment of wounds.—*Abstract of Medicine and Surgery.*

---

*On Salivary Fistula.*—In a recent number of the *Gazette des Hopitaux* (p. 814) is the account of a patient who was exhibited to the Academie de Medecine after the cure of a salivary fistula. He was a boy aged twelve, who fell in running down a staircase with a chamber-pot in his hand. His cheek was cut, and three fistulous openings formed—one corresponding to the situation of Sten'o duct, which showed no tendency to heal, the two others situated over the gland itself closed spontaneously. An artificial canal was formed through the cheek, by means of a trocar, and kept dilated with tents and bougies. When this canal had been completely established, the wound was closed by twisted suture. The cure at the time of exhibition [more than three months after the accident] seemed complete. During the presence of the fistula, Dr. Prompt, his medical attendant, made several observations on the boy which led him to the following conclusions.

1. The discharge of parotid saliva depends on the excitation of the sense of taste, and is pretty nearly the same whatever be the form of that excitation, whether the person eats or drinks, or a sapid substance is applied to the tongue.

2. The quantity of the liquid secreted depends chiefly on the time during which the excitation of the sense of taste lasts: thus, if a glass of wine is swallowed at once, only a few drops will be secreted; if it be drunk in small mouthfuls, so as to spend three or four minutes over it, a considerable quantity, as much as two or three grammes, will be obtained.—*London Med. Record.*

---

*Neuralgia of the Lower Jaw and Tongue treated by Excision of the Nerve.*—Dr. Theo. A. McGraw, of Detroit, reports the case of Mr. H., of Fairbault, Iowa, who had suffered for several years from a neuralgia of the left side of the tongue and lower jaw, which was becoming more and more constant and severe. The agony was so great that he was at times prevented from talking or eating. He had tried every remedy of which he had heard, and came to Ann Arbor, in the winter of 1871-72, to have the nerves leading to the painful areas excised. I saw him first in

January, 1872. I examined him carefully, to find, if possible, a cause for his terrible suffering. The teeth had been for the most part already extracted, and the gums seemed sound and natural. Careful investigation failed to reveal any eccentric cause of irritation, and I finally yielded to his desire, and operated upon him, in the hope that the disease might have its seat in the terminal nervous filaments. I performed the operation in the presence of the medical class, and by the assistance of Dr. Frothingham, on the third of February, 1872. I cut down upon the ramus of the lower jaw, splitting the masseter muscle and pushing its fibres aside. I then trephined the bone at the point where the inferior dental canal begins, and excised through the opening about half an inch of the inferior dental and gustatory nerves. The patient did well and recovered.

"I wrote to him a few weeks since to inquire as to the final result, and learned that he had remained free from pain for fourteen months. In April of 1873 it began to return, and now he describes it as bad as ever."

Dr. McGraw, in reviewing this case, says, "it is evident that the seat of the neuralgia is in this case somewhere in the peripheral nervous filaments, although no disease of the tongue or jaw could be discovered by the eye or touch. The recurrence of the pain in April, 1873, would indicate that a regeneration of the nervous tissue had taken place at that time, and that the nerves had then begun to resume their functions. I have advised the patient to have a tenotome passed through the jaw at the spot where it was trephined, and have the nerves re-divided. It would seem as if some process by which the nervous regeneration could be prevented in these cases ought to cure neuralgia of this description.

"I have been pleased with a suggestion of Dr. W. H, Stevens, of Fairbault, respecting neuralgias confined to the jaw alone. The doctor suggests that the dental canal be plugged wiih gold after the division of the nerve, thus making its regeneration impossible."—*Detroit Review of Med. and Phar.*

---

*Richardson's Tooth-Edge Cutting Scissors.*—These scissors are intended for the removal of tumors, and for general use in any operation where it is desirable to avoid much hæmorrhage. They are particularly suitable for cases of epulis. They are of the ordinary construction in all respects except in the cutting edge. The cutting edge of each blade, instead of being even and sharp is divided into finely-pointed teeth, each tooth being directed with a slight inclination towards the handle of the scissors. When the blades meet the teeth cross each other, and as they pierce any structure that may lie between them, they crush also, between their surfaces. If a piece of moderately firm substance be placed between the blades—a piece of paper or thin card, for example—the scissors perforate it in a series of perforations resembling what is seen in the postage stamp—that is to say, they do not cut clean through the substance so as to leave it in two distinct parts at once. A little lateral or half-rotating movement of the closed blades is, however, sufficient to tear through the still connected lines of substance and to complete the separation. The same occurs if the substance placed between the blades be a portion of soft animal structure, only that more force is required in the lateral or rotating movement to cause complete separation. The parts punctured are crushed between the teeth, and are separated by the twist or torsion. I find these scissors useful in dividing directly and quickly structures in which there are many minute blood-vessels, and which, when divided by the knife, bleed freely. These toothed scissors, as they can be made at one and the same time to pierce, crush, and twist, control bleeding remarkably.—*Lancet.*

---

*Disinfectants.*—Take a shallow and broad saucer and partly cover it with chloride of lime, then pour upon it a half ounce of impure carbolic acid. To fumigate rooms after death, to the above add a little strong sulphuric acid. Carbolic acid is not now regarded with so much favor as heretofore ; it is disagreeable and irritating to the lungs; it impregnates the food, and neighbors are annoyed by its odor.—*American Medical Weekly.*

---

*A Simple Plan of Ventilation.*—The following simple method of ventilating ordinary sleeping and dwelling-rooms is recommended by Mr. Hinton in his " Physiology for Practical Use :" " A Piece of wood, three inches high, and exactly as long as the breadth of the window, is to be prepared. Let the sash be now raised, the slip of wood placed on the sill, and the sash drawn closely upon it. If the slip has been well fitted, there will be no draft in consequence of this displacement of the sash at its lower part ; but the top of the lower sash will overlap the bottom of the upper one, and between the two bars perpendicular currents of air, not felt as draft, will enter and leave the room." —*Druggists Circular.*

---

*Moth Preventive*—The following recipe for keeping moths out of clothing is a favorite in some families : Mix half pint of alcohol, the same quantity of spirits of turpentine, and two ounces of camphor. Keep in a stone bottle, and shake before using. The clothes or furs are to be wrapped in linen, and crumpled-up pieces of blotting paper dipped in the liquid are to be placed in the box with them, so that it smells strong. This requires renewing about once a year.—*Druggists Circular.*

---

*Colored Inks.*—The following recipes have been well tested and are commended by good authorities as preferable to the solutions of aniline dyes which are now so extensively used as colored inks:—

*Green.* Two parts acetate of copper, one part carbonate of potash, and eight parts water. Boil till half is evaporated, and filter.

*Blue.* Three parts Prussian blue, one part oxalic acid, and thirty parts of water. When dissolved, add one part of gum-arabic.

*Yellow.* One part fine orpiment, well rubbed up with four parts thick gum-water.

*Red.* With the aid of a gentle heat, dissolve four grains of carmine in one ounce of aqua ammonia, and add six grains of gum-arabic.

*Gold.* Rub gold-leaf, such as is used by book-binders, with honey, till it forms a uniform mixture. When the honey has been washed out with water, the gold powder will settle at the bottom, and must be mixed with gum-water in sufficient quantity.

*Silver.* Silver-leaf treated in precisely the same manner gives a silver ink. Both these inks may, when dry, be polished with ivory.

*Black.* Three ounces crushed gall-nuts, two ounces crystallized sulphate of iron, two ounces gum arabic, and twenty-four ounces water.

*White.* Fine French zinc-white, or white-lead, rubbed up with gum-water to the proper consistency.—*Boston Jour. of Chemisty.*

---

*How to Remove Brass or Gold Rings from the Finger.*—Dr. Wm. Hauser recommends for the removal of gold rings the pouring of quicksilver over the ring. An amalgam is instantly formed, and the ring can be readily snapped by pressure with the fingers. To remove a brass ring, melted wax is poured all over the ring, enough to fill up all the gaps between the ridges on the afflicted finger. The wax is made to run under the finger also, to insure full protection to the skin. Then by a knife a small hole is made in the wax down to the ring, and muriatic acid poured into this hole. In a short time this will have combined with the elements of the brass. The ring can then be broken by pressure.—*Atlanta Med. Journal.*

*Nelaton's Method of Resuscitation from Chloroform Narcosis.*—Dr. I. Marion Sims (*British Medical Journal*, Aug. 22, 1874) most impressively details his observations respecting the practical workings of the above method. Briefly stated, it consists of in reversing the body of the patient, either by hanging him up by the feet or laying him over a bed or table, so that the blood from the greater part of the body can gravitate towards the head. The tongue is drawn forward by a tenaculum and artificial respiration tried. This practice is based on the theory that death from chloroform takes place from cerebral anæmia. —*Detroit Review of Medicine.*

---

*External Application of Iodine.*—Dr. Laborde strongly recommends M. Bouvier's formula for an iodine paint, to be applied externally. It consists of 30 parts of tincture of iodine, 2½ parts pure iodine, and 1½ parts of potassium iodide. M. Bouvier employs this paint in Pott's disease, along the sides of the spine and over the congestive abscesses, so frequently met with in this disease. But M. Laborde finds that it is most useful in serious effusions, as in simple hydrarthrosis, in rheumatic or traumatic effusion into joints, in hydrocele, etc. Even in simple pleuritic effusion it is very serviceable, not indeed in the early stage, but when this is confirmed. It may also be employed in certain congestive states of the viscera, especially in congestion of the liver, acute hypertrophic cirrhosis, and the accompanying ascites. He is accustomed to cover the painted part with a thick layer of cotton-wood; and he points out that it is easy to incorporate with it various calmative remedies, as morphia, opium, and belladonna. It then becomes very useful in relieving the pain of sciatica.—*Bulletin General de Therapeutique.*

---

*Passage of Arsenic and Antimony into the Tissues and Secretions.*—MM. Mayencon and Bergeret have discovered that arsenic and antimony can be readily detected by converting them into their hydrogen compounds and allowing the gases to come in contact with a piece of test paper dipped in perchloride of mercury. Arseniuretted hydrogen causes a citron yellow stain on the paper, and antimoniuretted hydrogen a greyish-brown stain. They detect arsenic in the urine of a person taking it by putting about an ounce of the morning urine in a flask with a piece of pure zinc, pure sulphuric acid, and a little sugar candy, and then placing the paper soaked in mercurial solution on the mouth of the flask. By this method they find that arsenic is rapidly absorbed, and appears in the urine immediately. It is some time before its elimination is completed. When no more appears in the urine, but some remains in the body, sulphurous waters aid its complete expulsion. Antimony is absorbed and diffused in the organism more slowly, and its elimination by the urine rarely begins on the first day, and generally only does so after it has been given for several days. Experiments on animals show that arsenic and antimony are eliminated both by the liver and kidneys, but chiefly by the liver.—*La France Medicale.*

---

*Weight of Men and Women.*—At the late Franklin Institute Exhibition there were weighed 15,840 men, aggregating 2,314, 260 pounds; 17,437 women, aggregating 2,249,370; making the average weight of each man 149¾ pounds, and of each woman 129 pounds.

---

*Anæsthesia by Injection of Chloral into the Veins.*—Professors Deneffe and Van Wetter report seven new cases in which operations have been successfully performed under the anæsthesia produced by the injection of chloral. They state that this plan has now been successfully tried in eighteen cases, and they give the details of the last seven, one of which was by Oré, of Bordeaux, the originator of the plan.

Oré's case was a young man, twenty years of age, from whom a tuberculous testicle was to be removed. The steps of the operation were substantially as follows: It was commenced at 8.43 A. M., at which time the median basilic vein was punctured and one minute later 30 grains of chloral had been injected; after two minutes the patient appeared sleepy; the pulse was 112. At 8.51 A. M., there was complete insensibility, and the operation was commenced, lasting seventeen minutes. At 8.59 A. M., there appeared to be some difficulty in breathing, from the relaxation of the tongue, which blocked up the posterior portion of the buccal cavity. By raising the hyoid bone and passing a current over the left hypogastric, this condition was overcome. The amount of chloral injected was about 100 grains, and the injection lasted seven minutes, producing complete anæsthesia, which lasted about an hour.

The patient remained in a deep sleep fourteen and a half hours, during which time his sensibility was very obtuse. The respiration was always quiet and uniform, and there was no vomiting and no period of excitement. No irritation was observed at the place where the vein was punctured.

Another case was of a woman, forty-eight years of age, who was operated on for entropion. In this case 77 grains of chloral were injected into the median basilic vein, at the rate of about 7 grains every half minute. In four minutes and twenty-five seconds complete anæsthesia of the cornea had been obtained with about 60 grains of chloral. Complete anæsthesia lasted for forty-seven minutes, and sleep for seven hours. The results of the operation were also excellent in this case, and, on the second day following, the patient was walking in the garden. The writers observe that in all these eighteen cases no serious disturbances of respiration have been noticed, which some have claimed take place when this experiment is tried on dogs. In one case, where the patient was very athletic, 150 grains of chloral were given in thirteen and a half minutes. There was no accident in this case, though the first urine passed was found to contain albumen and blood, so that really a slight hæmaturia was produced, but this passed away and the patient did well. The writers remark that they observed less embarrassment in respiration when the solution of chloral was pretty fluid. They have generally used the solution in the strength of one to four of water, but they believe that when it was still more diluted the respiration and circulation were less affected.—*Bull. del' Acad. Roy. de Med. de Belgique.*

---

*Eucalyptus and Tobacco.*—A captain of the French army, according to "Science Pour Tous," not being able to abandon the use of tobacco for smoking, although its use caused him serious inconvenience, has for several years mixed a few dried leaves of the eucalyptus globulus with the portion of the weed which he is in the habit of using daily. Since he has employed this mixture, he asserts that he has experienced neither vertigo, headache, nor pain in the stomach. He recommends this means for preventing the disagreeable effects of the pipe, without compelling the individual to renounce his old habit.—*Medical and Surgical Reporter.*

---

*American Medical Degrees.*—These are getting more and more out of repute in Great Britain through the sale of diplomas from institutions in this country which claim the privilege of issuing them. It appears, from recent journals, that a man has been fined, in Dumfriesshire, for appending his name with the letters M. D., to a certificate of death. The man produced a diploma from the *Livingstone University* in America, and the *Edinburgh University of Chicago.* [Are there any such institutions having a legal existence?] The *Lancet* observes (Nov. 21), "it is well known that degrees in America are as plentiful as mushrooms." . . . For the prosecution it was shown that Dr. Robertson, secretary to one of the colleges in question, and who is at present in London, had offered to grant a degree from the same university as that from which the defendant held his diploma for £10, without the necessity of a course of study, and without examination.—*Med. News and Library.*

